# A Comprehensive Machine Learning Approach for COVID-19 Target Discovery in the Small-Molecule Metabolome

**DOI:** 10.3390/metabo15010044

**Published:** 2025-01-11

**Authors:** Md. Shaheenur Islam Sumon, Md Sakib Abrar Hossain, Haya Al-Sulaiti, Hadi M. Yassine, Muhammad E. H. Chowdhury

**Affiliations:** 1 Department of Electrical Engineering, Qatar University, Doha P.O. Box 2713, Qatar; sumon@qu.edu.qa; 2 Department of Biochemistry, University of Regina, Regina, SK S4S 0A2, Canada; mah690@uregina.ca; 3 Department of Biomedical Sciences, College of Health Sciences, Qatar University, Doha P.O. Box 2713, Qatar; haya.alsulaiti@qu.edu.qa; 4 Biomedical Research Center, Qatar University, Doha P.O. Box 2713, Qatar

**Keywords:** metabolomics, respiratory viruses, machine learning, diagnostic markers, COVID-19

## Abstract

**Background/Objectives:** Respiratory viruses, including Influenza, RSV, and COVID-19, cause various respiratory infections. Distinguishing these viruses relies on diagnostic methods such as PCR testing. Challenges stem from overlapping symptoms and the emergence of new strains. Advanced diagnostics are crucial for accurate detection and effective management. This study leveraged nasopharyngeal metabolome data to predict respiratory virus scenarios including control vs. RSV, control vs. Influenza A, control vs. COVID-19, control vs. all respiratory viruses, and COVID-19 vs. Influenza A/RSV. **Method:** We proposed a stacking-based ensemble technique, integrating the top three best-performing ML models from the initial results to enhance prediction accuracy by leveraging the strengths of multiple base learners. Key techniques such as feature ranking, standard scaling, and SMOTE were used to address class imbalances, thus enhancing model robustness. SHAP analysis identified crucial metabolites influencing positive predictions, thereby providing valuable insights into diagnostic markers. **Results:** Our approach not only outperformed existing methods but also revealed top dominant features for predicting COVID-19, including Lysophosphatidylcholine acyl C18:2, Kynurenine, Phenylalanine, Valine, Tyrosine, and Aspartic Acid (Asp). **Conclusions:** This study demonstrates the effectiveness of leveraging nasopharyngeal metabolome data and stacking-based ensemble techniques for predicting respiratory virus scenarios. The proposed approach enhances prediction accuracy, provides insights into key diagnostic markers, and offers a robust framework for managing respiratory infections.

## 1. Introduction

Globally, the coronavirus disease 2019 (COVID-19) pandemic caused widespread disruptions and a substantial loss of human lives. SARS-CoV-2, the virus that causes COVID-19, enters the human body primarily via nasal epithelial cells [1]. The primary immune reaction to the virus occurs within a distinct immune microenvironment known as the nasopharynx-associated lymphoid tissue system, which is located in the nasal cavity [1]. Influenza, a widespread illness affecting both humans and animals, is caused by viruses that have animal reservoirs and exhibit continuous antigenic change [2]. Both COVID-19 and Influenza are contagious respiratory illnesses [3]. COVID-19 spreads through respiratory droplets, aerosols, and contaminated surfaces [3]. Influenza, caused by Influenza A or B viruses, spreads primarily through respiratory droplets released during coughing or sneezing [4]. Respiratory syncytial virus (RSV) is also a highly contagious virus causing acute respiratory infections, with a global incidence of approximately 33 million cases in children under 5 years; RSV infection often leads to severe bronchiolitis [5]. Molecular testing, specifically polymerase chain reaction (PCR), has revolutionized the surveillance and diagnosis of infectious diseases in clinical microbiology and virology laboratories over the past decade [6,7]. Although these techniques are rapid and accurate, they continue to have notable limitations, including cost, complicated procedure, inability to differentiate active infection from latency or colonization and diminished sensitivity when applied to direct patient specimens [6,7,8].

With varying degrees of success, the application of the “omics” method, comprising genomics, proteomics, and metabolomics, has been investigated for diagnosing COVID-19 and Influenza [9,10,11,12,13,14]. In contrast to conventional clinical virology diagnostics, metabolomics, which examines small molecules on a large scale, identifies the metabolic response of the host rather than explicitly identifying the pathogen [15]. Alterations in the nasal metabolome that are specific to a particular virus have been observed to correlate with viral load and disease severity [16].

The COVID-19 pandemic has underscored the urgent need for reliable tools to predict disease severity, improve diagnosis, and guide treatment strategies. Advanced proteomic and metabolomic profiling, combined with machine learning, has emerged as a powerful approach to uncovering molecular alterations in COVID-19 patients, enabling the identification of critical biomarkers [17]. Techniques such as TMT-labeled proteomics, UPLC-MS/MS, and LC-MS-based metabolomics have successfully stratified severe and non-severe cases by analyzing proteins, metabolites, and lipid profiles [17,18]. These studies have revealed changes like increased creatinine levels and reduced arginine/kynurenine ratios, as well as metabolic shifts in lysophosphatidylcholines (LPCs) and phosphatidylcholines (PCs) that evolve with disease progression and revert to baseline upon recovery [18,19]. Metabolomics facilitates early diagnosis, risk stratification, and disease monitoring when integrated with machine learning algorithms, achieving diagnostic specificity exceeding 96% and sensitivity over 83% [18]. However, significant challenges remain, including high costs, the complexity of high-dimensional data analysis, variability in sampling phases, incomplete longitudinal data due to patient discharge or mortality, and limited representation of newer SARS-CoV-2 variants [19,20]. Addressing these limitations through large-scale, diverse cohort studies is essential to validate these promising biomarkers and ensure their clinical applicability.

Bennet et al. [16] utilized a targeted metabolomics approach based on LC-MS/MS to analyze nasopharyngeal swabs from patients infected with SARS-CoV-2, Influenza A (INFA), Respiratory Syncytial Virus (RSV), and healthy controls. The study aimed to identify characteristic changes in the nasal metabolome of infected patients to discover significant metabolites relevant to pathogenicity and potential therapeutic targets. A total of 210 individuals were included in the study, divided into four groups: 55 COVID-19 patients (SARS-CoV-2 positive), 55 Influenza A patients, 56 RSV patients, and 44 unaffected controls. Using liquid chromatography-tandem mass spectrometry (LC-MS/MS), the researchers quantified 141 analytes from the viral transport media (VTM) of nasopharyngeal swabs. Utilizing their dataset, we proposed a comprehensive machine learning approach for metabolomics profiling. Instead of relying on individual models, we implemented stacking-based ensemble techniques that combined probabilities from the top three initial models. To ensure explainability, SHAP (SHapley Additive exPlanations) was employed to assess the contributions and impacts of top features on the model. The contributions of this study are as follows:

○ We proposed stacking-based ensemble learning, which was applied with five-fold cross-validation utilizing the publicly available LC–MS/MS dataset of the nasopharyngeal metabolome of COVID-19;
○ Top features were selected using the Random Forest algorithm, and statistical analyses such as the chi-square test, T-test, and Ranksum test were performed;
○ The proposed method was applied to the following classification scenarios: (A) Control vs. RSV, (B) Control vs. Influenza A, (C) Control vs. COVID-19, (D) Control vs. All respiratory viruses, and (E) COVID-19 vs. Influenza A/RSV, to discover significant metabolites in each case;
○ SHAP analysis was used to evaluate the contribution of significant features in each case to identify the most important metabolites.

The rest of the paper is organized as follows: Section 2 presents the related works; Section 3 describes the methodology, including the dataset description, models, and statistical analysis; Section 4 provides the results and discussion; and Section 5 concludes the paper with the conclusion.

## 2. Related Works

Machine learning (ML) has become a powerful tool for navigating the intricacies of metabolomics data, thus facilitating efficient analysis, interpretation, and extraction of valuable insights [21,22,23,24]. Recently, Kantz et al. [25] have created, fine-tuned, and evaluated an ML pipeline that effectively classifies spectral features in non-targeted liquid chromatography–mass spectrometry (LC/MS) metabolomics data by using both deep neural networks and a simpler multiple Logistic Regression model. Jeany et al. [26] introduced a novel approach that integrates mass spectrometry and ML using paired m/z analysis for direct COVID-19 diagnosis from raw data. This method presents a flexible tool for population screening and risk assessment in public health initiatives, addressing ion competition effects and compatible with a range of mass spectrometers, such as flow-injection mass spectrometry. This technique offers molecular insights into the pathogenesis of COVID-19, with potential uses for managing patients during the pandemic and other related disorders.

Metabolomics and ML strategies have the potential to revolutionize the diagnosis of infectious diseases, specifically respiratory viruses. Hogan et al. [27] have applied liquid chromatography quadrupole time-of-flight (LC/Q-TOF) and ML for Influenza diagnosis based on nasopharyngeal swab samples. After an initial analysis of 236 samples, the researchers extended their approach to a clinically applicable LC/MS analysis in a cohort of 96 symptomatic individuals. Hasan et al. [28] have applied metabolomics strategies for analyzing volatile organic compounds in exhaled breath and using mass spectrometry for COVID-19 detection in nasopharyngeal swabs. The study highlights the differentiation between targeted and untargeted approaches, thus stressing the need for standardization and extensive clinical validation before the integration of volatile organic compound-based tests into clinical practice. Recently, Bennet et al. [16] have systematically examined the nasopharyngeal metabolome in patients with COVID-19 using a liquid chromatography tandem mass spectrometry (LC–MS/MS) kit, quantifying 141 analytes. Through qRT-PCR and the use of ML models, the study [16] has achieved remarkable accuracy in discerning viral infections, specifically distinguishing COVID-19 from other respiratory viruses, and identifying critical differentiating metabolites in the process. Juan et al. [29] most recently employed machine learning (ML) and explainable artificial intelligence (XAI) to analyze metabolic alterations in COVID-19 and post-COVID-19 patients, revealing heterogeneous metabolic responses. In a cohort of 142 COVID-19, 48 post-COVID-19, and 38 control patients, the study outperformed traditional methods such as PCA and PLS-DA by utilizing XGBoost enhanced with SHAP values. Valuable insights into disease progression and long-term metabolic impacts were obtained by identifying key metabolites, including taurine, glutamine, and LysoPC a C16:0. In a separate recent study [30], Maryne et al. demonstrated that the prognostication and triage performance of COVID-19 patients can be substantially enhanced by the combination of high-definition metabolomics and machine learning (ML). In total, 64 PCR-positive COVID-19 patients underwent an analysis of their metabolomics profiles and clinical parameters using high-resolution mass spectrometry. While the AUC of standard clinical parameters (SpO_2_, respiratory rate, Horowitz quotient, and age) was 0.85 for predicting severity (need for mechanical ventilation), the prediction performance was significantly enhanced by the incorporation of metabolomics data, resulting in an AUC of 0.92.

## 3. Methods

This section contains a detailed explanation of the methods used to identify respiratory viruses in small-molecule metabolomes including the dataset, preprocessing methods, and model implementation.

**Figure 1:**
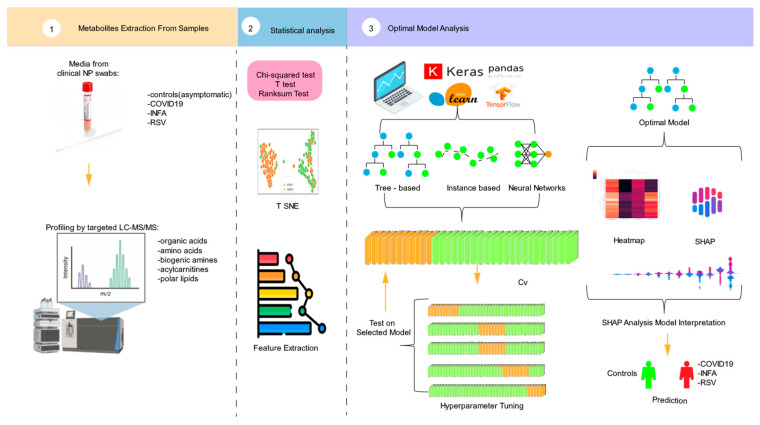
Graphical depiction of the experimental structure.

Figure 1 provides an outline of the workflow process. The investigation began with an analysis of clinical nasopharyngeal swabs, obtained from a publicly available dataset, using a viral transport medium (VTM) and a TMIC Prime kit (The kit was acquired from The Metabolomics Innovation Centre (TMIC), located in Edmonton, Alberta, Canada). This procedure involved chemical derivatization and LC–MS/MS. Statistical analyses were conducted, incorporating *p*-values, chi-square tests, and t-distributed stochastic neighbor embedding t-SNE plots. Subsequently, a feature extraction process was executed, wherein the top ten feature ranks were identified. A 5-fold dataset was generated to facilitate robust model training. Various ML models were used, including tree-based models, instance-based models, and neural networks. The stacking ensemble technique was applied to create an optimal model for predicting the final output. To further elucidate the influential metabolites associated with specific respiratory viruses, we conducted a SHAP analysis. This analytical approach was aimed at identifying and quantifying individual metabolites on the predictive models, thereby contributing to a comprehensive understanding of the metabolomic landscape in relation to respiratory virus presence.

### 3.1. Dataset Description

The dataset was reported by Bennet et al. [16], who conducted a study using nasopharyngeal specimens from individuals infected with COVID-19, Influenza A, and RSV, along with unaffected controls. Using an LC–MS/MS-based screening system to quantify 141 analytes, the nasopharyngeal metabolome was characterized. SARS-CoV-2 positive, Influenza A positive, and RSV positive patients comprised the remaining 210 members of the dataset. Individuals were classified into unaffected controls and three distinct patient groups. A thorough examination of the metabolomic distinctions between various respiratory viruses and control subjects was achieved by analysis of the small-molecule profiles in viral transport medium extracted from nasal samples from each group. The demographic characteristics of all patients are presented in Supplementary Table S1, including essential information, such as the number of individuals, collection year (including monthly variation), age range, sex distribution expressed as a percentage, and median computed tomography attenuation (CTa) with the corresponding range. A comprehensive list of all metabolites assessed in the study, along with their detailed information, is documented in Supplementary Table S2.

**Figure 2:**
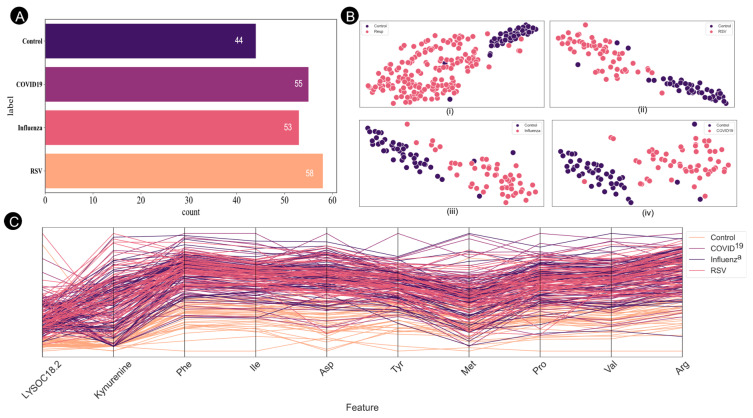
(**A**) Numbers of samples in the affected and control groups. (**B**) Graphical representation of the ten most highly ranked features in the feature space, as determined by the ranking model using the selected random forest feature. Respiratory virus samples are represented in red, while control samples are represented in pink. (i) Control vs. all respiratory viruses. (ii) Control vs. RSV. (iii) Control vs. Influenza. (iv) Control vs. COVID-19. (**C**) Parallel coordinate plot illustrating class separability for ten selected feature spaces.

Figure 2 illustrates the comprehensive analysis of the dataset, including both the total sample distribution and patient classes. The t-distributed stochastic neighbor embedding (t-SNE) [31] plot visually depicts the distinct class separations, and provides insights into the clustering patterns for both the control group and individual respiratory virus categories. Additionally, a parallel coordination plot is presented, highlighting the class separability across the top ten features for the four identified classes. This integrated approach provides a thorough examination of the dataset, combining descriptive statistics, dimensionality reduction, and feature visualization to enhance understanding of the underlying patterns and relationships within the data.

### 3.2. Statistical Analysis

A statistical analysis in Python 3.9 was performed to evaluate the central values of the features and the distribution of the data. The significance of individual features in relation to the objective variable was determined with *p*-values calculated with a variety of statistical tests, such as the chi-square test, Wilcoxon rank-sum test, and T test [32,33].

In the initial state, the dataset comprised 48 metabolite features. Through the implementation of a stringent feature selection method, the ten most promising features were identified. The following section provides an in-depth analysis of their specific implications. The notable characteristics are outlined in Table 1, which presents a comparative statistical analysis between the control group and the group of all respiratory viruses for the top 10 features. These features include Lysophosphatidylcholine 18:2 (LysoPC 18:2), Kynurenine (Kyn), Phenylalanine (Phe), Isoleucine (Ile), Aspartic Acid (Asp), Tyrosine (Tyr), methionine sulfoxide (Met.SO), proline (Pro), valine (Val), and arginine (Arg).

### 3.3. Dataset Preprocessing

The dataset used in this study was originally reported by Bennet et al. To enhance the efficacy of ML models during training, normalization of the input data was necessary, to ensure that each feature contributed proportionately, thereby improving overall model performance. In this context, the Standard Scaler method was used for normalization [34,35]. To promote robust training and facilitate generalization, the dataset was subjected to a 5-fold cross-validation, involving partitioning the data into training and testing sets (80% and 20%, respectively). This strategic data-splitting method aided in assessing model performance across different subsets of the dataset and contributed to a more reliable evaluation of the model’s ability to generalize to unseen data.

To address the class imbalance within the dataset, wherein the counts for RSV, COVID-19, Influenza, and control classes were 58, 55, 53, and 44, respectively, the pipeline used Synthetic Minority Over-sampling Technique (SMOTE) augmentation [36]. This technique helps mitigate the effects of imbalanced class distribution during training by generating synthetic samples for the minority classes. By oversampling the minority classes, SMOTE contributes to a more balanced representation across all classes, enhancing the model’s ability to effectively learn from and generalize to each class during the training process.

Feature ranking is an essential preemptive measure in the field of ML [37], particularly when datasets comprise a large number of features. This method is critical to prevent overfitting, which occurs when a model overly adjusts to the complexities of the training data, thereby impairing its performance when applied to novel datasets. For five separate investigations, the XBGoost, Random Forest, and ExtraTrees algorithms were used to rank the 48 features. The Random Forest algorithm initially ranked highest, surpassing the performance of the other two approaches.

### 3.4. Classification Model Development

In our experiment, we used MLP Classifier, ElasticNet, Linear Discriminant Analysis, XGBoost Classifier, Random Forest Classifier, Logistic Regression, ExtraTrees Classifier, AdaBoost Classifier, KNN Classifier, and Gradient Boosting Classifier. The top-performing models are described below.

#### 3.4.1. Random Forest Classifier

The Random Forest (RF) [38] Classifier is a machine learning algorithm designed for classification tasks. It is an ensemble method that makes predictions by combining the outputs of multiple decision trees. The name “random forest” originates from its process of building a “forest” of decision trees, each created randomly. These trees are formed by determining the best splitting points in the data, often using metrics like Gini impurity or information gain. Unlike traditional decision trees that consider all features at each split, the Random Forest algorithm selects the split points from a randomly chosen subset of features at each node.

#### 3.4.2. Linear Discriminant Analysis

Linear Discriminant Analysis (LDA) [39] is a dimensionality reduction method and supervised classification technique. It identifies a linear combination of features that most effectively distinguishes between two or more classes by maximizing the between-class variance and minimizing the within-class variance. This is especially beneficial for datasets that have distributed classes that are well separated. LDA functions by projecting the data into a lower-dimensional space while preserving the most discriminative information. It is mathematically elegant and computationally efficient because it presupposes that the data in each class is normally distributed and shares the same covariance matrix.

#### 3.4.3. XGBoost Classifier

XGBoost [40] is a highly efficient and scalable tree-boosting system widely used for achieving state-of-the-art results in machine learning tasks. A key challenge in tree learning is identifying optimal split points, which traditionally involves an exact greedy algorithm that exhaustively evaluates all possible splits across features. However, this approach becomes computationally expensive, particularly for continuous features. To address this, XGBoost optimizes the process by sorting data based on feature values. By processing the sorted data in ascending order, the algorithm efficiently accumulates gradient statistics, which are essential for determining the optimal split and enhancing the structure score. This ingenious strategy significantly reduces the computational burden while maintaining accuracy.

#### 3.4.4. Logistic Regression

Logistic Regression [41] is a statistical model that is frequently employed for binary and multi-class classification assignments. It applies the sigmoid function to a linear combination of input features to estimate the probability that a class belongs to a given input. Logistic Regression is frequently employed as a baseline model in machine learning due to its effectiveness in linearly separable data, despite its simplicity. It presupposes a linear relationship between the log-odds of the objective variable and the input features. The model is a popular choice in disciplines such as finance, social sciences, and medical research due to its robustness to small datasets, interpretability, and computational efficiency. By preventing overfitting, regularization techniques like L1 (Lasso) and L2 (Ridge) can further enhance its performance.

#### 3.4.5. ExtraTreesClassifier

ExtraTreesClassifier [42] (Extremely Randomized Trees) is a collaborative learning approach that enhances the accuracy and robustness of classification by constructing multiple decision trees. ExtraTrees, in contrast to Random Forest, incorporates additional randomness during tree construction by randomly selecting split points for each feature and subsequently selecting the best-performing split. This preserves predictive performance and enhances computational efficiency while reducing model variance. ExtraTrees is notably effective on high-dimensional data and noisy datasets due to its randomness, which renders it resistant to overfitting. It is frequently employed for tasks that necessitate the evaluation of feature importance, the identification of outliers, and the classification of structured datasets.

#### 3.4.6. KNeighborsClassifier

KNeighborsClassifier (K-Nearest Neighbors) [43] is a non-parametric, instance-based learning algorithm that is designed for classification tasks. It functions by employing a distance metric, typically Euclidean distance, to identify the K nearest neighbors of a data point in the feature space. The class is then assigned based on a majority vote among the neighbors. KNN is straightforward, and intuitive, and does not necessitate an explicit training phase, as predictions are generated from the stored dataset. The efficacy of the algorithm is contingent upon the data structure, the distance metric, and the selection of *K*. Although KNN is computationally expensive for large datasets, it is effective for smaller datasets and when the decision boundaries are non-linear.

#### 3.4.7. ElasticNet

For high-dimensional data with correlated features, ElasticNet [44] is a regularized regression method that effectively incorporates both L1 (Lasso) and L2 (Ridge) penalties. ElasticNet ensures model stability (via L2) while performing feature selection (via L1) by balancing L1 and L2 penalties. By distributing the weight among groups of correlated features, this method circumvents Lasso’s limitations, which include the ability to select only one feature from a set of highly correlated features. In domains such as genomics, finance, and healthcare, ElasticNet is frequently employed in classification and regression problems where overfitting is a concern.

#### 3.4.8. Stacking Ensemble Approach

In our experiment, we proposed a stacking-based ensemble technique instead of relying on a single ML model. We selected the top 3 best-performing ML models from the initial results and integrated them into a stacking framework. Stacking combines multiple base learners (classical ML models) to leverage their individual strengths, thereby enhancing overall prediction accuracy. This ensemble approach significantly improves the model’s predictive capabilities compared to using individual models. Stacking is an ensemble learning technique that combines the predictions of numerous base models to enhance forecasting precision [45,46]. The initial phase involved training individual ML models subsequently, the top three performing models were selected according to their predictive capabilities. Notably, a random forest was chosen as the meta model. The core of this technique involves using the meta-model to acquire and combine information from many base models, thus enhancing prediction ability. The use of stacking, as exemplified by Rahman et al. [47], has produced noteworthy results in evaluation metrics, continually surpassing 90% in all assessment criteria.

A comprehensive probability distribution is constructed by combining predictions from the base-level classifier set N with the input variable x.

(1) $$P^{N}\left(x\right)=\left(P^{N}\left(c_{1}|x\right),P^{N}\left(c_{2}|x\right),\ldots \ldots ,P^{N}\left(c_{m}|x\right)\right)$$

**Figure 3:**
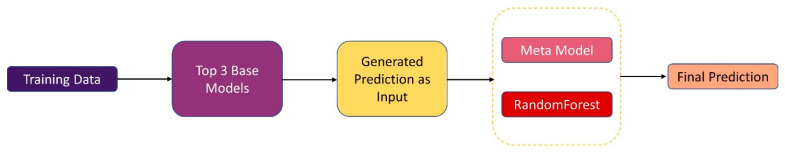
Stacking ensemble technique to combine base models and a meta-model.

The set of potential class values is represented as (c, $c_{2}\ldots$ $c_{m}$), and the probability that example y belongs to class bi, as determined and forecasted by classifier M, is given as P N(bi |x). Figure 3 illustrates a stacking-based approach where RandomForest serves as the meta-model. The prediction probabilities generated by the top 3 base models are combined as input to the meta-model, which then processes these probabilities to produce the final prediction.

### 3.5. Evaluation Metrics

The performance of the classifiers was assessed with receiver operating characteristic (ROC) curves and the area under the curve (AUC), as well as precision, sensitivity, specificity, accuracy, and F1-Score. Furthermore, we used a five-fold cross-validation technique, which involved splitting the dataset into 80% for training and 20% for testing. This process was repeated five times to validate the complete dataset, on the basis of the fold number. We used per-class weighted metrics and overall precision, because of the varying number of instances across classes. Furthermore, the AUC value was used as an assessment criterion. The mathematical representation of five evaluation measures (weighted sensitivity or recall, specificity, precision, total accuracy, and F1 score) can be found in Equations (2)–(6).

(2) $$Accuracy_{class\_x}=\frac{TP_{class\_x}+TN_{class\_x}}{TP_{class\_x}+TN_{class\_x}+FP_{class\_x}+FN_{class\_x}}$$

(3) $$Precision_{class\_x}=\frac{TP_{class\_i}}{TP_{class\_x}+FP_{class\_x}}$$

(4) $$Recall/Sensitivity_{class_{x}}=\frac{TP_{class_{i}}}{TP_{class_{x}}+FN_{class_{x}}}$$

(5) $$F1\_score_{class_{x}}=2\frac{Precision_{class_{x}}\times Sensitivity_{class_{i}}}{Precision_{class_{x}}+Sensitivity_{class_{x}}}$$

(6) $$Specificity_{class\_x}=\frac{TN_{class\_x}}{TN_{class\_x}+FP_{class\_x}}\;\;\;\;\;$$

Here, the terms “true positive,” “true negative”, “false positive”, and “false negative” are abbreviated as *TP*, *TN*, *FP*, and *FN*, respectively.

### 3.6. Model Explainability

The ability to comprehend and interpret the decisions or predictions generated by an ML model, referred to as “explainability”, encompasses a range of methods and strategies that reveal the process through which a model derives its outcomes, thereby enhancing the model’s transparency. SHAP [48], a method for explaining models that measure the individual effect of each attribute on the model’s prediction, offers valuable information regarding how specific characteristics affect the output of the model, thus improving the comprehensibility and clarity of intricate ML models.

## 4. Results and Discussion

This section includes the following: (i) feature ranking, (ii) detailed outcomes of the top-performing model, (iii) results pertaining to model explainability, and (iv) a comprehensive discussion and comparative analysis. This structured presentation is aimed at providing a nuanced understanding of the study’s outcomes and their implications.

### 4.1. Feature Ranking

**Figure 4:**
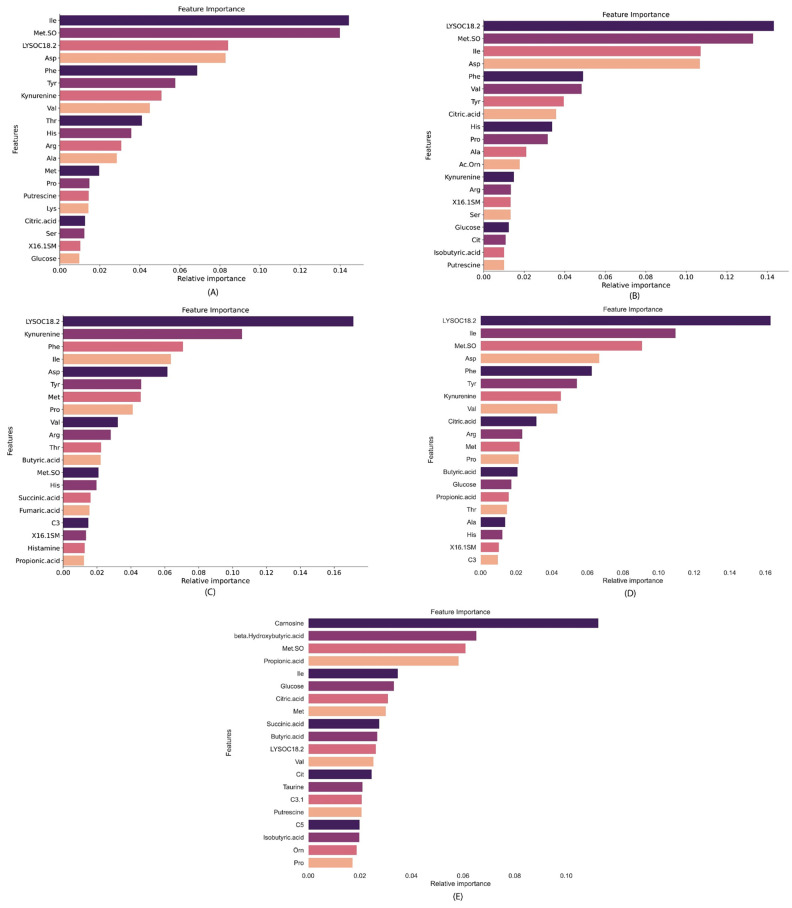
Top features ranked with the random forest feature selection algorithm (**A**) control vs. RSV (**B**), control vs. Influenza, (**C**) control vs. COVID-19, (**D**) control vs. all respiratory virus, and (**E**) COVID-19 vs. Influenza A/RSV.

In this investigation, three advanced ML feature selection models—XGBoost, random forest, and extra trees—were used. After a thorough preliminary exploration, the random forest model was found to exhibit superior performance, achieving the highest rankings. From the initial set of 48 features, the top ten features emerged as particularly impactful, delivering optimal results with a minimal subset of features. Figure 4 indicates the top features, ranked through the random forest feature selection algorithm, across distinct comparisons: (A) control vs. RSV, (B) control vs. Influenza, (C) control vs. COVID-19, (D) control vs. all respiratory virus, and (E) COVID-19 vs. Influenza/RSV. These visual representations offer a concise provision of insight into the discriminative power of selected features in differentiating among the specified conditions.

### 4.2. Classification Model Results

The comprehensive evaluation process comprised five distinct scenarios: control vs. RSV, control vs. Influenza A, control vs. all respiratory viruses, COVID-19 vs. all respiratory viruses, and Influenza A/RSV. In the initial phase, 13 variants of machine learning models were trained using a five-fold dataset. The three best models selected from the initial stage were used to provide probabilities as inputs for training the meta-models in the stacking-based ensemble.

**Figure 5:**
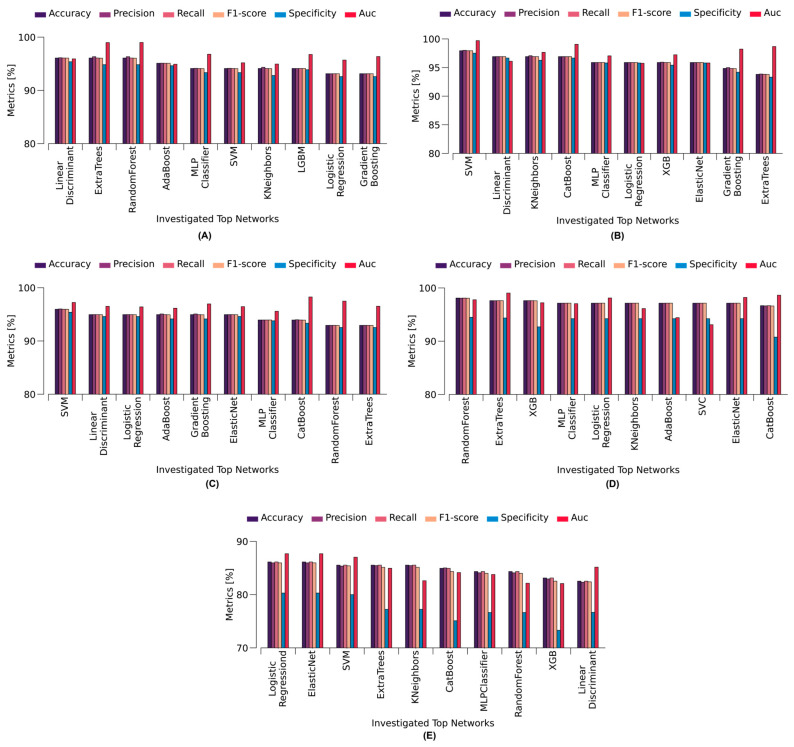
Top performing stacking-based models for different cases: (**A**) control vs. RSV, (**B**) control vs. Influenza A, (**C**) control vs. COVID-19, (**D**) control vs. all respiratory virus, and (**E**) COVID-19 vs. Influenza A/RSV.

The application of stacking achieved notable improvements in the evaluation metrics, particularly for scenarios involving control vs. all respiratory viruses and COVID-19 vs. all Influenza A/RSV. However, for the remaining scenarios, no improvement in metrics was observed. Figure 5 visually presents the top ten performing stacking-based models across the five scenarios, providing a clear and concise overview of the models’ performance in each distinct case.

Figure 5A indicates the outcomes for the control vs. RSV scenario, with linear discriminant analysis emerging as the top-performing model. Demonstrating superior performance across various evaluation metrics, this model achieved an accuracy of 96.08%, precision of 96.13%, recall of 96.08%, specificity of 95.38%, F1-score of 96.07%, and an AUC of 95.92%. Figure 5B reveals the exceptional performance of SVM as the leading model in the control vs. Influenza A scenario. SVM outperformed other models, with an accuracy of 97.94%, precision of 98.01%, recall of 97.94%, specificity of 97.51%, F1-score of 97.93%, and an impressive AUC of 99.69%. In Figure 5C, the control vs. COVID-19 scenario highlights SVM as the preeminent model, exhibiting an accuracy of 95.96%, precision of 96.02%, recall of 95.96%, specificity of 95.4%, F1-score of 95.95%, and AUC of 97.23%. Figure 5D reveals random forest as the top performer in the control vs. all respiratory virus scenario, achieving an exceptional 98.1% accuracy, 98.09% precision, 98.1% recall, 94.48% specificity, F1 score of 98.08%, and an AUC of 97.78%. In Figure 5E, Logistic Regression emerges as the superior performer in the COVID-19 vs. Influenza A/RSV scenario, with commendable metrics, including an accuracy of 86.14%, precision of 85.97%, recall of 86.14%, specificity of 80.3%, F1 score of 85.97%, and an AUC of 87.68%. Notably, the lower accuracy in this case was attributed to the class imbalance issue for COVID-19, with 55 samples, compared with 110 samples for Influenza A/RSV.

Further detailed results for each case are presented in Supplementary Table S3 to Supplementary Table S7. These tables illustrate both the initial results and the detailed performance of the stacking-based models. A clear improvement in predictive accuracy is observed with the stacking-based approach, with at least a 1% enhancement in performance compared to the initial models, as evidenced by the data in these tables. The confusion matrices and AUC curves for the top-performing models in each scenario are presented, as illustrated in Supplementary Figures S1 and S2.

### 4.3. Model Explainability According to Shap Values

SHAP [49] helps understand the impact of each feature on the model’s output for a particular prediction, offering valuable insights into the model’s decision-making process. This method uniquely highlights the individual contribution of each feature towards a specific prediction, thereby providing a nuanced understanding of the global and local behaviors inherent in the model. By emphasizing transparency and elucidating the decision-making process, SHAP is aimed at instilling trust in the ML approach among end-users. SHAP not only enhances interpretability but also promotes a more informed and confident engagement with the model’s predictions.

**Figure 6:**
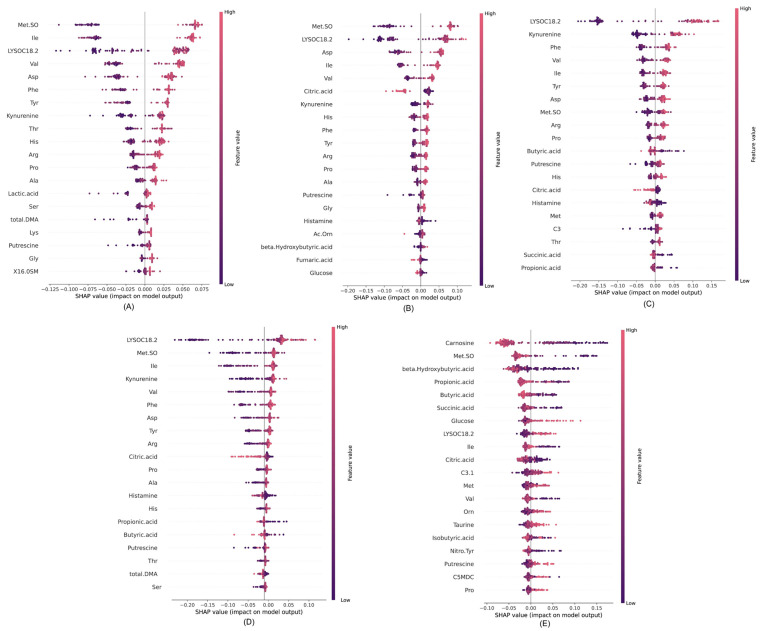
SHAP values for different cases: (**A**) control vs. RSV, (**B**) control vs. Influenza A, (**C**) control vs. COVID-19, (**D**) control vs. all respiratory viruses, and (**E**) COVID-19 vs. Influenza A/RSV.

We conducted SHAP analysis in three distinct scenarios for our research, considering all relevant attributes. Figure 6 demonstrates the impact of SHAP values on the model outputs across these scenarios. The horizontal axis represents the direction of the effect, with positive impacts indicated by red and negative impacts by blue. In this context, red corresponds to higher feature values, while blue corresponds to lower feature values. SHAP can identify the significant features or metabolites with their corresponding impacts.

In Figure 6A, for the control vs. RSV scenario, the SHAP analysis highlights distinct feature effects on model predictions. Specifically, Met.SO (Methionine sulfoxide) had a substantial positive effect on RSV predictions, indicative of the higher concentrations in RSV cases than control. Notably, Ile, Val, Asp, Phe, and showed considerable positive effects, thus emphasizing their influential roles in predicting RSV cases. In Figure 6B, focusing on the control vs. Influenza A scenario, the SHAP analysis revealed LYSOC18:2 as the predominant metabolite feature with the greatest effect on predicting Influenza A cases. In Figure 6C for control vs. COVID-19, LYSOC18:2 again emerges as the dominant feature, in agreement with previous findings by Bennet et al. [16], thereby establishing its value in distinguishing COVID-19 cases. Other notable metabolite features, including Kynurenine, Phe, Val, Tyr, and Asp, contributed significantly to the predictive model. For the control vs. all respiratory virus scenario, as depicted in Figure 6D, LYSOC18:2 was the most dominant feature, thus indicating its crucial role in discriminating cases involving respiratory viruses collectively.

Finally, in the control vs. RSV/Influenza A scenario represented in Figure 6E, Carnosine emerged as the most dominant feature for predicting COVID-19 cases. This detailed analysis provided valuable insights into the specific metabolite features driving the predictive capability of the model across various respiratory virus classification scenarios.

### 4.4. Discussion

Respiratory viruses, including Influenza A, RSV, and COVID-19, pose major health challenges [50,51,52]. Our work focused on leveraging LC/MS-MS metabolomics data to predict the presence of respiratory viruses in individuals, by discerning dominant metabolites, contributing to accurate classification. Applying a similar method to various diseases allowed us to explore distinct metabolite profiles and gain insights into the underlying biochemical dynamics across different pathological conditions. ML models can discern complex patterns within the data [53] and identify subtle metabolic changes associated with specific viral infections. This approach enables a more nuanced understanding of disease dynamics.

A comprehensive statistical analysis was conducted for control, normal, and all respiratory virus scenarios, by using chi-square tests, rank sum tests, and T-tests. Twenty ML models were trained for five distinct scenarios: control vs. RSV, control vs. Influenza A, control vs. COVID-19, control vs. all respiratory viruses, and COVID-19 vs. Influenza A/RSV. Feature ranking techniques were applied to select the top ten features. Standard scaling was used to normalize the data, and a five-fold dataset was created. Before model fitting, the SMOTE was used to address class imbalance.

Among the 13 variants of ML models, the top ten performers were selected, and a stacking ML model was trained by using the three most successful models. The outcomes of each model are illustrated in Figure 5. Notably, linear discriminant analysis excelled in the control vs. RSV scenario, whereas SVM stood out in the control vs. Influenza A scenario. The control vs. COVID-19 and control vs. all respiratory virus scenarios indicated SVM and random forest as the leading models, respectively. Logistic Regression emerged as the superior performer in the COVID-19 vs. Influenza A/RSV scenario.

Furthermore, the SHAP value analysis provides a detailed understanding of feature importance in distinguishing various respiratory conditions. In the control vs. RSV case, Met.SO, Ile, and LYSOC18.2 emerge as dominant biomarkers, with Met.SO showing the most substantial positive impact. For control vs. Influenza A, similar patterns are observed, where Met.SO and LYSOC18.2 remain critical, alongside Asp, Kynurenine, and Phe, indicating their significant roles in identifying Influenza A. In differentiating control vs. COVID-19, LYSOC18.2 and Kynurenine stand out as key features, while metabolites like Phe, Val, and Ile also contribute notably. When comparing control vs. all respiratory viruses, the features LYSOC18.2, and Met.SO consistently demonstrate their importance across multiple cases, with contributions from Kynurenine, Ile, and Citric Acid, reinforcing their significance in detecting respiratory infections. Finally, in distinguishing COVID-19 vs. Influenza A/RSV, Carnosine emerges as a unique and dominant biomarker, with additional impacts from Met.SO and beta-Hydroxybutyric acid, highlighting its role in differentiating COVID-19 from other viral infections. Overall, Met.SO, LYSOC18.2, and Kynurenine repeatedly prove to be critical biomarkers across multiple conditions, while Carnosine demonstrates unique utility in identifying COVID-19 specifically, showcasing the distinct metabolic profiles associated with different respiratory viruses.

We utilized a stacking-based ensemble technique to improve predictive performance, rather than relying on a single machine learning model. The stacking approach utilizes the prediction probabilities from the top three best-performing models identified during the initial evaluation phase. These models were selected based on their superior accuracy and other evaluation metrics. By integrating them into a stacking framework, we aimed to combine the individual strengths of the base learners (classical machine learning models), thus enhancing the overall predictive accuracy. Furthermore, we conducted a comparative analysis of the proposed stacking-based approach with and without the use of the Synthetic Minority Over-sampling Technique (SMOTE) to address the class imbalance. The comparison in Table 2 highlights the effectiveness of our proposed stacking-based ensemble technique compared to traditional supervised machine learning models. Bennett et al. [16] achieved notable performance with supervised methods; however, their models showed limitations in specificity and sensitivity. The random forest model with SMOTE outperformed its counterpart without SMOTE, achieving higher accuracy (98.10% vs. 96.67%), sensitivity (98.10% vs. 96.66%), and specificity (94.48% vs. 92.44%), demonstrating the importance of addressing the class imbalance. Logistic Regression showed moderate results, with significant declines in performance without SMOTE. Our stacking-based ensemble, which integrates prediction probabilities from the top three best-performing models, consistently improved accuracy and sensitivity, particularly when paired with SMOTE, outperforming individual models. These findings emphasize the robustness of stacking combined with SMOTE for enhanced predictive performance.

## 5. Conclusions

This study concludes that leveraging machine learning with nasopharyngeal metabolome data effectively distinguishes the control group from various respiratory viral infections, including COVID-19, RSV, and Influenza A. We proposed a stacking-based ensemble technique that integrates the top three best-performing machine learning models, enhancing prediction accuracy by leveraging the strengths of multiple base learners. Using a combination of stacking, feature selection, and SMOTEs significantly improved model performance, achieving superior accuracy, sensitivity, and specificity. Statistical analyses, including rank sum tests, T-tests, and chi-square tests, were performed to identify significant metabolites. Metabolites such as Met.SO, LYSOC18.2, and Kynurenine emerged as critical biomarkers across multiple conditions, while Carnosine uniquely distinguished COVID-19 from other viral infections. SHAP analysis provided explainable insights into feature contributions, identifying key metabolites influencing positive predictions and reinforcing the clinical potential of metabolomics and machine learning for accurate diagnostics. Notably, our approach outperformed existing methods and revealed dominant features for predicting COVID-19, including Lysophosphatidylcholine acyl C18:2, Kynurenine, Phenylalanine, Valine, Tyrosine, and Aspartic Acid (Asp), which are essential in metabolic pathways. Our XAI (Explainable AI) analysis further proposed the top significant features for each respiratory virus case, demonstrating the robustness and interpretability of our model in identifying key diagnostic markers.

## Figures and Tables

**Table 1:** Statistical analysis of the characteristics of the metabolite features (control vs. all respiratory viruses).

Control vs. All Respiratory Viruses					
Feature Name	Control	Respiratory Virus	Total	Technique	*p*-Value
Sex Male (%) Female (%) Null (%)	25% 75% 0%	42.77% 47.59% 9.63%	53.33% 39.04% 7.62%	Chi-square test	<0.05
LYSOC18.2 Mean ± SD Median	0.86 ± 1.05 0.8725	1.57 ± 0.97 1.4427	1.42 ± 1.03 1.2314	Rank-sum test	<0.0001
Ile Mean ± SD Median	19.57 ± 15.78 15.50	69.76 ± 42.48 66.90	59.24 ± 43.54 53.30	Rank-sum test	<0.0001
Met.SO Mean ± SD Median	1.27 ± 1.97 0.5445	6.74 ± 6.29 5.90	5.59 ± 6.08 5.02	Rank-sum test	<0.0001
Asp Mean ± SD Median	54.54 ± 25.06 49.350	139.60 ± 58.74 132.50	121.78 ± 63.70 116.00	T-test	<0.0001
Phe Mean ± SD Median	24.54 ± 16.97 21.40	85.80 ± 44.40 84.05	72.97 ± 47.33 70.40	Rank-sum test	<0.0001
Tyr Mean ± SD Median	23.24 ± 12.52 22.60	72.33 ± 43.25 62.95	62.04 ± 43.70 54.90	T-test	<0.0001
Kynurenine Mean ± SD Median	3.88 ± 2.72 6.224	6.85 ± 7.05 5.190	6.22 ± 6.50 5.3550	Rank-sum test	0.0067
Val Mean ± SD Median	32.43 ± 29.98 26.250	122.04 ± 89.86 111.00	103.26 ± 88.86 85.85	Rank-sum test	<0.0001
Citric acid Mean ± SD Median	3.26 ± 1.68 3.840	1.76 ± 4.21 1.070	2.08 ± 3.86 1.28	T-test	0.02169
Arg Mean ± SD Median	42.75 ± 24.27 36.150	134.68 ± 73.22 132.00	v115.42 ± 75.90 92.75	Rank-sum test	<0.0001

**Table 2:** Comparison of evaluation metrics with other studies.

	Model	Cases	Accuracy	Sensitivity	Specificity
Bennet et al. [16]	Supervised machine learning	Control vs. all respiratory virus	96%	98%	86%
		COVID-19 vs. influenza A/RSV	85%	74%	90%
Stacking-Based Ensemble Approach	RandomForest (With SMOTE)	Control vs. all respiratory virus	98.10%	98.10%	94.48%
	RandomForest (Without SMOTE)		96.67	96.66	92.44
	Logistic Regression (With SOMOTE)	COVID-19 vs. influenza A/RSV	86.14%	86.14%	80.3
	Logistic Regression (Without SMOTE)		84.94	84.94	77.86

## Data Availability

The dataset used in this study can be accessed at https://github.com/ColauttiLab/COVID-Metabolomics (accessed on 23 may 2023).

## Supplementary Materials

The following supporting information can be downloaded at: https://www.mdpi.com/article/10.3390/metabo15010044/s1, Figure S1: AUC curve for different cases; Figure S2: Confusion Matrix curve for different cases; Table S1: Demographic characteristics of all patients; Table S2: List of metabolites; Table S3: Top performed models results for Control vs. RSV; Table S4: Top performed models results for Control vs. Influenza A; Table S5: Top performed models results for Control vs. Covid 19; Table S6: Top performed models results for Control vs. All Respiratory Virus; Table S7: Top performed models results for COVID-19 vs. influenza A/RSV.
