# Supplementary material for: A Comprehensive Machine Learning Approach for COVID-19 Target Discovery in the Small-Molecule Metabolome
Source: Metabolites. 2025 Jan 11;15(1):44. doi: 10.3390/metabo15010044 (PMC11767724; doi:10.3390/metabo15010044)
Supplement: Supplementary file 1 [file metabolites-15-00044-s001.zip › metabolites-3289447-supplementary.pdf]

# Supplementary Materials

**Supplementary Table S1:** Demographic characteristics of all patients.

| | Patient class | | | |
|---|---|---|---|---|
| | SARS-COV-2 (COVID19) | Infuenza A (INFA) | Respiratory Syncytial Virus (RSV) | Controls |
| Number | 55 | 55 | 56 | 44 |
| Collection year (monthly variation) | 2020 (Jan–Apr) | 2019–2020 (Dec–Mar) | 2019–2020 (Dec–Mar) | 2020 (Sept) |
| Age range | 55 years (20–85 years) | 62 years (27 days–94 years) | 16 months (21 days– 91 years) | 24 years (19–43 years) |
| Sex (%) | M 27%<br>F 42%<br>n/a 31% | M 54%<br>F 44%<br>n/a 2% | M 40%<br>F 46%<br>n/a 14% | M 25%<br>F 75% |
| Median CTa (range) | 26.2 (17.76–37.24) | 28.6 (21.43–38.6) | 26.3 (18.54–36.87) | N/A |

**Supplementary Table S2:** List of metabolites

| **Acylcarnitines** | | | |
|---|---|---|---|
| C0 | Carnitine | C10:1 | Decenoylcarnitine |
| C2 | Acetylcarnitine | C10 | Decanoylcarnitine |
| C3:1 | Propenoylcarnitine | C12:1 | Dodecenoylcarnitine |
| C3 | Propionylcarnitine | C12 | Dodecanoylcarnitine |
| C4:1 | Butenylcarnitine | C14:2 | Tetradecadienylcarnitine |
| C4 | Butyrylcarnitine | **C14:1**[1,2] | **Tetradecenoylcarnitine** |
| C3OH | Hydroxypropionylcarnitine | C14 | Tetradecanoylcarnitine |
| C5:1[3] | Tiglylcarnitine | C12DC | Dodecanedioylcarnitine |
| C5 | Valerylcarnitine | C14:2OH | Hydroxytetradecadienylcarnitine |
| C4OH | Hydroxybutyrylcarnitine | C14:1OH | Hydroxytetradecenoylcarnitine |
| C6:1 | Hexenoylcarnitine | C16:2 | Hexadecadienylcarnitine |
| C6 | Hexanoylcarnitine | C16:1 | Hexadecenoylcarnitine |
| C5OH | Hydroxyvalerylcarnitine | C16 | Hexadecanoylcarnitine |
| C5:1DC | Glutaconylcarnitin | C16:2OH | Hydroxyhexadecadienylcarnitine |
| C5DC | Glutarylcarnitine | **C16:1OH**[2] | **Hydroxyhexadecenoylcarnitine** |
| C8 | Octanoylcarnitine | C16OH | Hydroxyhexadecanoylcarnitine |
| C5MDC | Methylglutarylcarnitine | C18:2 | Octadecadienylcarnitine |
| C9 | Nonaylcarnitine | C18:1 | Octadecenoylcarnitine |
| C7DC | Pimelylcarnitine | C18 | Octadecanoylcarnitine |
| C10:2 | Decadienylcarnitine | C18:1OH | Hydroxyoctadecenoylcarnitine |
| **Amino Acids** | | | |

| | | | |
|---|---|---|---|
| **Ala**[1,2,3] | **Alanine** | **Lys**[1,2,3] | **Lysine** |
| Arg[1,3] | Arginine | **Met**[1,2,3] | **Methionine** |
| **Asn**[1,2] | **Asparagine** | **Orn**[1,2,3] | **Ornithine** |
| **Asp**[1,2,3] | **Aspartate** | **Phe**[1,2,3] | **Phenylalanine** |
| **Cit**[1,2] | **Citrulline** | **Pro**[1,2,3] | **Proline** |
| **Gln**[1,2] | **Glutamine** | **Ser**[1,2,3] | **Serine** |
| Glu | Glutamate | **Thr**[1,2,3] | **Threonine** |
| **Gly**[1,2,3] | **Glycine** | Trp | Tryptophan |
| **His**[1,2,3] | **Histidine** | **Tyr**[1,2,3] | **Tyrosine** |
| **Ile**[1,2,3] | **Isoleucine** | **Val**[1,2,3] | **Valine** |
| **Leu**[1,2] | **Leucine** | | |
| **Benzenoids** | | | |
| Tyramine | | Homovanillic acid | |
| Hippuric acid | | | |
| **Biogenic Amines** | | | |
| Ac-Orn | Acetylornithine | **Met-SO**[1,2,3] | **Methionine sulfoxide** |
| ADMA | Asymmetric dimethylarginine | PEA | Phenylethylamine |
| alpha-AAA | alpha-Aminoadipic acid | **Putrescine**[1,2,3] | **Putrescine** |
| c4-OH-Pro | cis-4-Hydroxyproline | **Sarcosine**[1,2] | **Sarcosine** |
| Carnosine[3] | Carnosine | Serotonin | Serotonin |
| **Creatinine**[1,2] | **Creatinine** | **Spermidine**[1,2] | **Spermidine** |
| Dopamine | Dopamine | **Spermine**[1,2] | **Spermine** |
| **Histamine**[1,2,3] | **Histamine** | **t4-OH-Pro**[1,2] | **trans-4-Hydroxyproline** |
| **Kynurenine**[1,2,3] | **Kynurenine** | **Taurine**[1,2] | **Taurine** |
| Methylhistidine | Methylhistidine | **total DMA**[1,2] | **Dimethylamine** |
| **Glycerophospholipids** | | | |
| **LysoPC a C14:0**[1,2] | **LysoPhosphatidylcholine acyl C14:0** | LysoPC a C28:1 | LysoPhosphatidylcholine acyl C28:1 |
| **LysoPC a C16:1**[1,2] | **LysoPhosphatidylcholine acyl C16:1** | LysoPC a C28:0 | LysoPhosphatidylcholine acyl C28:0 |
| **LysoPC a C16:0**[1,2] | **LysoPhosphatidylcholine acyl C16:0** | PC aa C32:2 | Phosphatidylcholine diacyl C32:2 |
| **LysoPC a C17:0**[1,2] | **LysoPhosphatidylcholine acyl C17:0** | PC ae C36:0 | Phosphatidylcholine acyl-alkyl C36:0 |
| **LysoPC a C18:2**[1,2,3] | **LysoPhosphatidylcholine acyl C18:2** | PC aa C36:6 | Phosphatidylcholine diacyl C36:6 |
| **LysoPC a C18:1**[1,2] | **LysoPhosphatidylcholine acyl C18:1** | PC aa C36:0 | Phosphatidylcholine diacyl C36:0 |
| **LysoPC a C18:0**[1,2] | **LysoPhosphatidylcholine acyl C18:0** | PC aa C38:6 | Phosphatidylcholine diacyl C38:6 |
| LysoPC a C20:4 | LysoPhosphatidylcholine acyl C20:4 | Pc aa C38:0 | Phosphatidylcholine diacyl C38:0 |
| **LysoPC a C20:3**[2] | **LysoPhosphatidylcholine acyl C20:3** | Pc ae C40:6 | Phosphatidylcholine acyl-alkyl C40:6 |
| LysoPC a C24:0 | LysoPhosphatidylcholine acyl C24:0 | PC aa C40:6 | Phosphatidylcholine diacyl C40:6 |
| LysoPC a C26:1 | LysoPhosphatidylcholine acyl C26:1 | PC aa C40:2 | Phosphatidylcholine diacyl C40:2 |
| LysoPC a C26:0 | LysoPhosphatidylcholine acyl C26:0 | PC aa C40:1 | Phosphatidylcholine diacyl C40:1 |
| **Lipids and lipid-like molecules** | | | |
| Butyric acid | | | |
| **Organic acids and derivatives** | | | |
| **Betaine**[2] | | Citric acid[3] | |
| **Creatine**[2] | | Succinic acid[3] | |
| Diacetylspermine | | **Fumaric acid**[1,2] | |
| **Lactic acid**[1,2,3] | | Pyruvic acid | |
| **beta-Hydroxybutyric acid**[1,2,3] | | Isobutyric acid | |
| alpha-Ketoglutaric acid | | Methylmalonic acid | |
| DOPA | l-3,4-dihydroxyphenylalanine | Nitro-Tyr | Nitrotyrasine |

| | | | |
|---|---|---|---|
| Propionic acid | | p-Hydroxyhippuric acid | para-hydroxyhippuric acid |
| **Organoheterocyclic compounds** | | | |
| Indole acetic acid | | **Uric acid[1,2]** | |
| **Organic nitrogen compounds** | | | |
| **Choline[1,2]** | **Choline** | TMAO | Trimethylamine N-oxide |
| **Hexose** | | | |
| Glucose[3] | | | |
| **Phenylpropanoids and polyketides** | | | |
| HPHPA | 3-(3-Hydroxyphenyl)-3-hydroxypropanoic acid | | |
| Sphingomyelins | | | |
| SM(OH) C14:1 | Hydroxysphingomyeline C14:1 | SM C18:0 | Sphingomyeline C18:0 |
| SM C16:1 | Sphingomyeline C16:1 | SM C20:2 | Sphingomyeline C20:2 |
| **SM C16:0[1,2,3]** | **Sphingomyeline C16:0** | SM(OH) C22:2 | Hydroxysphingomyeline C22:2 |
| SM(OH) C16:1 | Hydroxysphingomyeline C16:1 | SM(OH) C22:1 | Hydroxysphingomyeline C22:1 |
| SM C18:1 | Sphingomyeline C18:1 | SM(OH) C24:1 | Hydroxysphingomyeline C24:1 |

**Supplementary Table S3:** Top performed models results for Control Vs RSV.

| Initial Results | | | | | | | Stacking Results | | | | | | |
|---|---|---|---|---|---|---|---|---|---|---|---|---|---|
| **Classifier** | **Acc** | **P** | **R** | **SP** | **F1** | **AUC** | **Classifier** | **Acc** | **P** | **R** | **SP** | **F1** | **AUC** |
| **ExtraTreesClassifier** | 95.1 | 95.22 | 95.1 | 94.09 | 95.08 | 98.94 | **LinearDiscriminantAnalysis** | 96.08 | 96.13 | 96.08 | 95.38 | 96.07 | 95.92 |
| **LGBM** | 95.1 | 95.11 | 95.1 | 94.63 | 95.09 | 98.15 | ExtraTrees | 96.08 | 96.33 | 96.08 | 94.83 | 96.05 | 98.98 |
| **LinearDiscriminant Analysis** | 94.12 | 94.12 | 94.12 | 93.89 | 94.12 | 95.65 | RandomForest | 96.08 | 96.33 | 96.08 | 94.83 | 96.05 | 99 |
| MLPClassifier | 93.14 | 93.13 | 93.14 | 92.6 | 93.13 | 95.92 | AdaBoost | 95.1 | 95.11 | 95.1 | 94.63 | 95.09 | 94.9 |
| RandomForest | 93.14 | 93.13 | 93.14 | 92.6 | 93.13 | 98.96 | MLPClassifier | 94.12 | 94.15 | 94.12 | ` | 94.09 | 96.78 |
| SVM | 93.14 | 93.13 | 93.14 | 92.6 | 93.13 | 94.86 | SVM | 94.12 | 94.15 | 94.12 | 93.34 | 94.09 | 95.18 |
| CatBoost | 93.14 | 93.13 | 93.14 | 92.6 | 93.13 | 98.86 | KNeighbors | 94.12 | 94.34 | 94.12 | 92.8 | 94.08 | 94.94 |
| KNeighbors | 92.16 | 92.18 | 92.16 | 91.31 | 92.13 | 94.14 | LGBM | 94.12 | 94.12 | 94.12 | 93.89 | 94.12 | 96.74 |
| XGB | 92.16 | 92.5 | 92.16 | 92.95 | 92.19 | 93.94 | LogisticRegression | 93.14 | 93.13 | 93.14 | 92.6 | 93.13 | 95.68 |
| AdaBoost | 92.16 | 92.26 | 92.16 | 92.4 | 92.17 | 94.9 | GradientBoosting | 93.14 | 93.13 | 93.14 | 92.6 | 93.13 | 96.35 |

**Supplementary Table S4:** Top performed models results for Control Vs Influenza A.

| Initial Results | | | | | | | Stacking Results | | | | | | |
|---|---|---|---|---|---|---|---|---|---|---|---|---|---|
| **Classifier** | **Acc** | **P** | **R** | **SP** | **F1** | **AUC** | **Classifier** | **Acc** | **P** | **R** | **SP** | **F1** | **AUC** |
| **LinearDiscriminantAnalysis** | 96.91 | 96.92 | 96.9 | 96.66 | 96.91 | 96.09 | **SVM** | 97.94 | 98.01 | 97.94 | 97.51 | 97.93 | 99.69 |
| **SVM** | 96.91 | 96.92 | 96.9 | 96.66 | 96.91 | 95.49 | LinearDiscriminantAnalysis | 96.91 | 96.92 | 96.9 | 96.66 | 96.91 | 96.09 |
| **CatBoost** | 96.91 | 96.92 | 96.9 | 96.66 | 96.91 | 99.05 | KNeighbors | 96.91 | 97.07 | 96.91 | 96.27 | 96.9 | 97.66 |

| | | | | | | | | | | | | |
|---|---|---|---|---|---|---|---|---|---|---|---|---|
| KNeighborsClassifier | 96.91 | 97.07 | 96.91 | 96.27 | 96.9 | 97.66 | CatBoost | 96.91 | 96.92 | 96.9 | 96.66 | 96.91 | 99.05 |
| MLPClassifier | 95.88 | 95.88 | 95.88 | 95.8 | 95.88 | 97.04 | MLPClassifier | 95.88 | 95.88 | 95.88 | 95.8 | 95.88 | 97.04 |
| XGB | 95.88 | 95.94 | 95.87 | 95.42 | 95.87 | 97.23 | LogisticRegression | 95.88 | 95.88 | 95.88 | 95.8 | 95.88 | 95.75 |
| ElasticNet | 95.88 | 95.88 | 95.88 | 95.8 | 95.88 | 95.79 | XGB | 95.88 | 95.94 | 95.87 | 95.42 | 95.87 | 97.23 |
| GradientBoosting | 94.85 | 94.99 | 94.84 | 94.18 | 94.82 | 98.22 | ElasticNet_untuned | 95.88 | 95.88 | 95.88 | 95.8 | 95.88 | 95.79 |
| ExtraTreesClassifier | 93.81 | 93.87 | 93.82 | 93.32 | 93.8 | 98.67 | GradientBoosting | 94.85 | 94.99 | 94.84 | 94.18 | 94.82 | 98.22 |
| RandomForest | 93.81 | 93.81 | 93.81 | 93.71 | 93.81 | 98.67 | ExtraTrees | 93.81 | 93.87 | 93.82 | 93.32 | 93.8 | 98.67 |

**Supplementary Table S5:** Top performed models results for Control Vs Covid 19.

| Initial Results | | | | | | | Stacking Results | | | | | | |
|---|---|---|---|---|---|---|---|---|---|---|---|---|---|
| **Classifier** | **Acc** | **P** | **R** | **SP** | **F1** | **AUC** | **Classifier** | **Acc** | **P** | **R** | **SP** | **F1** | **AUC** |
| **MLPClassifier** | 94.95 | 94.98 | 94.95 | 95.05 | 94.96 | 96.57 | **SVM** | 95.96 | 96.02 | 95.96 | 95.4 | 95.95 | 97.23 |
| **SVM** | 94.95 | 94.96 | 94.95 | 94.59 | 94.94 | 96.9 | LinearDiscriminant Analysis | 94.95 | 94.96 | 94.95 | 94.59 | 94.94 | 96.5 |
| **AdaBoost** | 94.95 | 95.08 | 94.95 | 94.14 | 94.93 | 95.99 | LogisticRegression | 94.95 | 94.96 | 94.95 | 94.59 | 94.94 | 96.4 |
| RandomForest | 94.95 | 94.96 | 94.95 | 94.59 | 94.94 | 97.37 | AdaBoost | 94.95 | 95.08 | 94.95 | 94.14 | 94.93 | 96.15 |
| CatBoost | 93.94 | 93.98 | 93.94 | 93.33 | 93.92 | 98.3 | GradientBoosting | 94.95 | 95.08 | 94.95 | 94.14 | 94.93 | 96.96 |
| LinearDiscriminantAnalysis | 93.94 | 93.98 | 93.94 | 93.33 | 93.92 | 96.36 | ElasticNet | 94.95 | 94.96 | 94.95 | 94.59 | 94.94 | 96.44 |
| LogisticRegression | 92.93 | 92.96 | 92.93 | 92.98 | 92.94 | 96.61 | MLPClassifier | 93.94 | 93.94 | 93.94 | 93.79 | 93.94 | 95.57 |
| ExtraTrees | 92.93 | 92.96 | 92.93 | 92.98 | 92.94 | 97.25 | CatBoost | 93.94 | 93.98 | 93.94 | 93.33 | 93.92 | 98.26 |
| LGBM | 92.93 | 92.96 | 92.93 | 92.98 | 92.94 | 96.77 | RandomForest | 92.93 | 92.93 | 92.93 | 92.53 | 92.92 | 97.47 |
| ElasticNet | 92.93 | 92.96 | 92.93 | 92.98 | 92.94 | 96.61 | ExtraTrees | 92.93 | 92.93 | 92.93 | 92.53 | 92.92 | 96.52 |

**Supplementary Table S6:** Top performed models results for Control Vs All Respiratory Virus.

| Initial Results | | | | | | | Stacking Results | | | | | | |
|---|---|---|---|---|---|---|---|---|---|---|---|---|---|
| **Classifier** | **Acc** | **P** | **R** | **SP** | **F1** | **AUC** | **Classifier** | **Acc** | **P** | **R** | **SP** | **F1** | **AUC** |
| **CatBoost** | 97.14 | 97.14 | 97.14 | 94.23 | 97.14 | 99.17 | **RandomForest** | 98.1 | 98.09 | 98.1 | 94.48 | 98.08 | 97.78 |
| **ExtraTrees** | 97.14 | 97.12 | 97.15 | 92.56 | 97.12 | 98.8 | ExtraTrees | 97.62 | 97.6 | 97.62 | 94.36 | 97.61 | 99.04 |
| **MLPClassifier** | 96.67 | 96.7 | 96.67 | 94.1 | 96.68 | 95.65 | XGB | 97.62 | 97.62 | 97.62 | 92.69 | 97.59 | 99.72 |
| AdaBoost | 96.67 | 96.8 | 96.67 | 95.77 | 96.7 | 96.71 | MLPClassifier | 97.14 | 97.14 | 97.14 | 94.23 | 97.14 | 97.04 |
| RandomForest | 96.67 | 96.64 | 96.66 | 92.44 | 96.65 | 98.8 | LogisticRegression | 97.14 | 97.14 | 97.14 | 94.23 | 97.14 | 98.12 |
| LogisticRegression | 96.19 | 96.28 | 96.19 | 93.98 | 96.22 | 96.13 | KNeighbors | 97.14 | 97.14 | 97.14 | 94.23 | 97.14 | 96.12 |
| SVM | 96.19 | 96.19 | 96.19 | 92.31 | 96.19 | 96.26 | AdaBoost | 97.14 | 97.14 | 97.14 | 94.23 | 97.14 | 94.42 |

| | | | | | | | | | | | | | |
|---|---|---|---|---|---|---|---|---|---|---|---|---|---|
| KNeighbors | 95.24 | 95.48 | 95.24 | 93.72 | 95.31 | 95.23 | SVM | 97.14 | 97.14 | 97.14 | 94.23 | 97.14 | 93.09 |
| LGBM | 95.24 | 95.24 | 95.24 | 90.39 | 95.24 | 98.41 | ElasticNet | 97.14 | 97.14 | 97.14 | 94.23 | 97.14 | 98.22 |
| ElasticNet | 95.24 | 95.48 | 95.24 | 93.72 | 95.31 | 96.15 | CatBoost | 96.67 | 96.64 | 96.67 | 90.77 | 96.62 | 98.64 |

**Supplementary Table S7:** Top performed models results for COVID-19 vs influenza A/RSV

| Initial Results | | | | | | | Stacking Results | | | | | | |
|---|---|---|---|---|---|---|---|---|---|---|---|---|---|
| **Classifier** | **Acc** | **P** | **R** | **SP** | **F1** | **AUC** | **Classifier** | **Acc** | **P** | **R** | **SP** | **F1** | **AUC** |
| **ExtraTrees** | 83.73 | 83.49 | 83.74 | 77.26 | 83.53 | 88.15 | **LogisticRegression** | 86.14 | 85.97 | 86.14 | 80.3 | 85.97 | 87.68 |
| **LogisticRegression** | 83.73 | 84 | 83.74 | 80.93 | 83.84 | 88.2 | ElasticNet | 86.14 | 85.97 | 86.14 | 80.3 | 85.97 | 87.68 |
| **ElasticNet** | 83.73 | 84 | 83.74 | 80.93 | 83.84 | 88.23 | SVM | 85.54 | 85.36 | 85.54 | 80 | 85.4 | 87.02 |
| RandomForest | 83.13 | 83 | 83.13 | 77.89 | 83.05 | 87.82 | ExtraTrees | 85.54 | 85.45 | 85.54 | 77.24 | 85.13 | 84.95 |
| LinearDiscriminantAnalysis | 82.53 | 82.61 | 82.53 | 78.51 | 82.57 | 85.2 | KNeighbors | 85.54 | 85.45 | 85.54 | 77.24 | 85.13 | 82.61 |
| CatBoost | 81.93 | 81.67 | 81.93 | 75.45 | 81.75 | 87.73 | CatBoost | 84.94 | 85.02 | 84.94 | 75.1 | 84.34 | 84.14 |
| SVM | 81.93 | 82.11 | 81.93 | 78.21 | 82.01 | 87.92 | MLPClassifier | 84.34 | 84.1 | 84.34 | 76.64 | 83.99 | 83.78 |
| KNeighbors | 80.72 | 81.69 | 80.72 | 79.44 | 81.02 | 82.07 | RandomForest | 84.34 | 84.1 | 84.34 | 76.64 | 83.99 | 82.13 |
| LGBM | 79.52 | 79.51 | 79.51 | 74.26 | 79.51 | 86.13 | XGB | 83.13 | 82.96 | 83.13 | 73.3 | 82.53 | 82.08 |
| MLPClassifier | 78.31 | 77.77 | 78.32 | 69.08 | 77.84 | 80.62 | LinearDiscriminant Analysis | 82.53 | 82.33 | 82.53 | 76.67 | 82.4 | 85.15 |

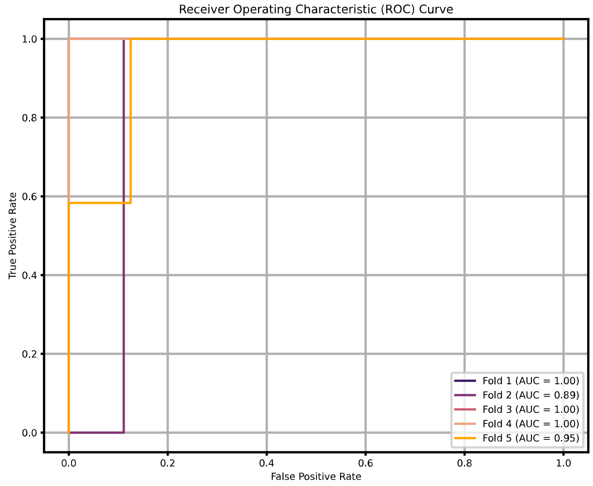


(A)

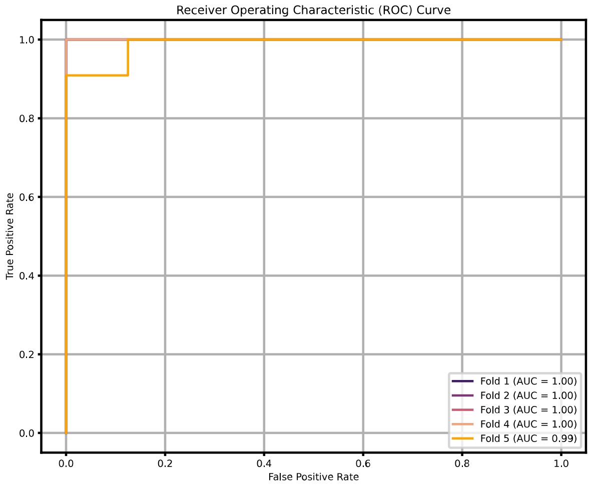


(B)

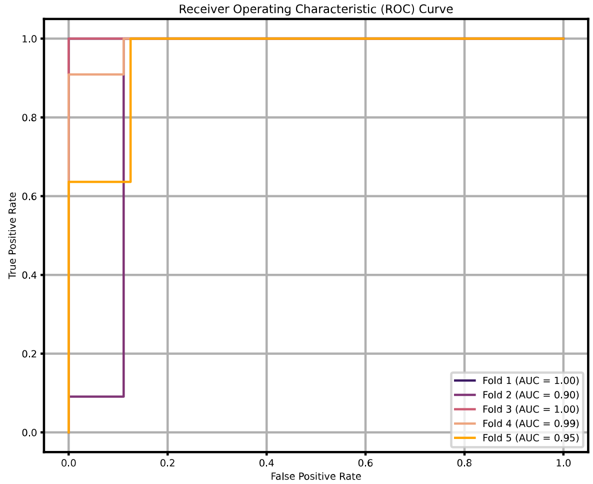


(C)

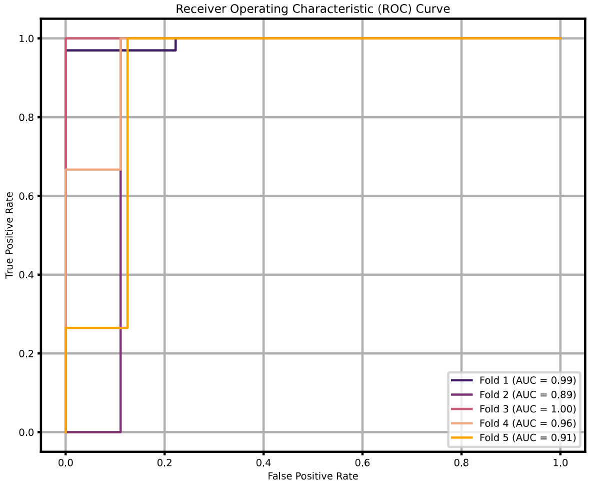


(D)

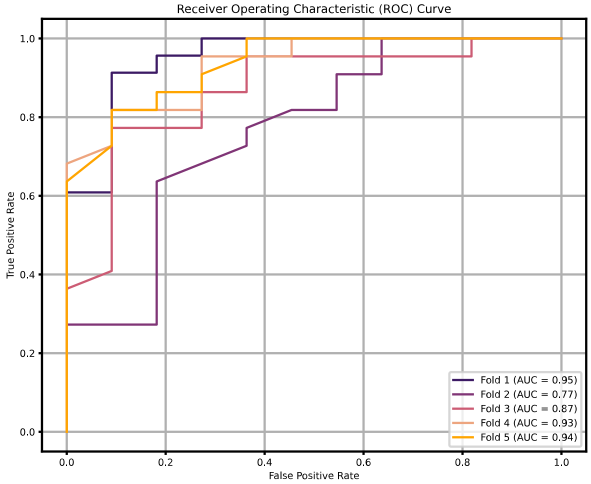


(E)

**Supplementary Figure S1:** AUC curve for different cases (A) Control Covid 19 Vs (Influenza A/RSV)Vs RSV (B) Control Vs Influenza A (C) Control Vs Covid 19 (D) Control Vs All Respiratory Virus (E) ) Covid 19 Vs (Influenza A/RSV).

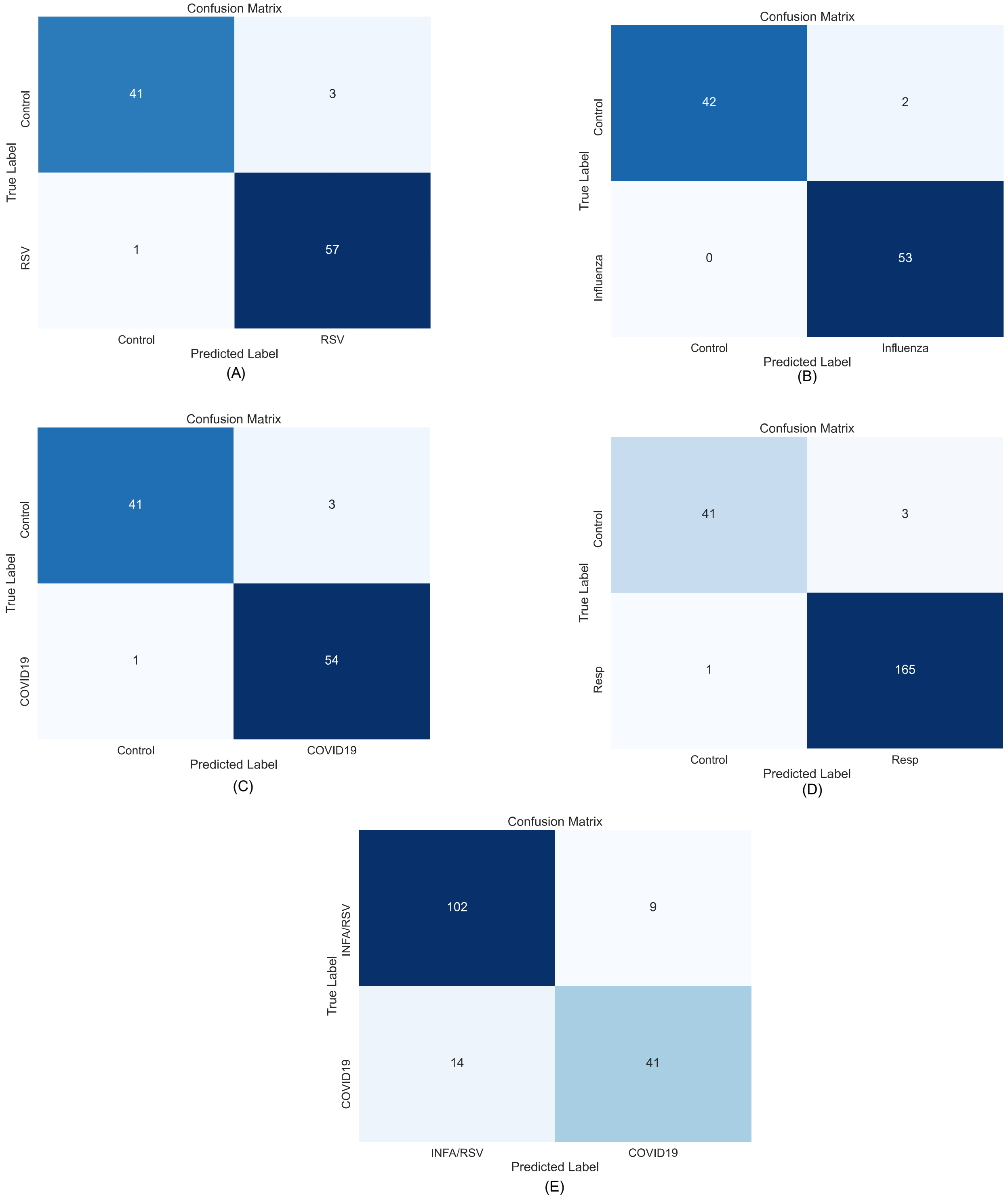


**Supplementary Figure S2:** Confusion Matrix curve for different cases (A) Control Covid 19 Vs (Influenza A/RSV)Vs RSV (B) Control Vs Influenza A (C) Control Vs Covid 19 (D) Control Vs All Respiratory Virus (E) ) Covid 19 Vs (Influenza A/RSV).
